# The Sick in Uncivilised Lands

**Published:** 1891-11-21

**Authors:** 


### The Doctor's Armchair.

### THE SICK IN UNCIVILISED LANDS.

Before the advent of railways, steamships, and the electric telegraph, the earth was felt to be a very large place by the majority of its inhabitants. It was only the astronomer, or some other like person, who was able to realise that, compared with all worlds, our little sphere was but a very small speck. Now, however, we are all beginning to feel conscious that India, China, Central Africa, and South America are quite within easy distance of us, whilst New York lies almost at our doors. To the homely worker, looking out for work, and to the ambitious man eager for a worthy career, this dwindling of the earth is an enlargement of opportunity.

It is clear to the observant eye that in England we have a great many strong and capable men who are tied down to inadequate careers. This is a statement which need not be argued. There are hundreds of military and civil servants of Her Majesty in India, Egypt, South Africa, and other places, who are doing splendid administrative work for weak and subject peoples, who would have been quite unknown and commonplace individuals if they had remained at home and without opportunity in England. It is opportunity which has made them what they are. Without such opportunity they would never have been more than what Carlyle calls "vague, wavering capabilities." They would have effected nothing either for themselves or for the world.

But notwithstanding the example of those who, by going abroad, have converted themselves into eminent and useful men, it is still the fact that large numbers of equally or even more capable persons stay at home and live in obscurity and comparative poverty, with their best faculties atrophied and dwarfed from want of opportunity and use. Conspicuous amongst these wasted stay-at-homes are a very great number of medical men. Those who are familiar with the higher class of English, Scotch, and Irish medical schools and students, have often been struck by the large number of handsome, well-built, bright, and clever fellows who throng the class-rooms. The medical student, it must be borne in mind, is a very free and expansive sort of young fellow. His mind is not limited by the artificial bounds of school logic: he is not reduced to timidity and feebleness of intellect by a too fanatical elementary religion: science enlarges his intellectual world, and with the enlargement he acquires a consciousness of strength and freedom. The medical student of the better class is undoubtedly a man of excellent mental capacity and of high courage.

What becomes of those hundreds of fine fellows who leave our British medical schools as fully qualified practitioners year after year? They have the capacity for doing great things. Of what sort are their actual careers? They ought to go where they are needed, and where they would be welcomed. If they did, their minds would continue to expand, and their manhood would become more weighty. They would acquire honourable positions and influence among their fellow men. Life would be energetic, successful, and happy for them. They would justify the promise of their boyhood. These are the things which might be; what are the things that are? Those bright young fellows hurry to make still larger a struggling crowd which is already twice too large. They remind one of the scenes which may be witnessed at the London Docks, when the gates are opened to admit those who are to be selected for the day's work, and when twenty men are eager to be employed where there is only work for one. The fact of twenty men dashing themselves upon employment for which one is sufficient is terrible enough when the men are uneducated labourers; it is infinitely worse when they belong to a class who have had a dozen or fifteen years of costly school and college life before they have entered upon the struggle for profitable work.

Is it really the fact that the world is over-peopled? Are there too many men of education for the work which requires to be done? Is it true, as Tennyson said so long ago in "Locksley Hall," that "Every gate is thronged with suitors; all the markets overflow"? It is not true. Some of the markets, perhaps a good many, do overflow. But since the time when Tennyson wrote "Locksley Hall," the markets of the world, the markets for both rough and cultured labour, have almost doubled. The competition for rough and cultured work has by no means increased at anything like the same rate. Is it not the case that the educated portion of these islands consists of men of strong and courageous intellect, who can force their way in difficult places, who can lead and govern men of weaker stamp? The greater part of the world is peopled by men of weaker stamp than the cultured Englishman. The larger part of Asia and nearly all Africa need to be infused with new life and to be directed in the pathways of intellectual and material development. Those continents are dying of physical and moral inanition; our cultured classes are suffocating each other by crowding together in such a little country as England, where there is hardly space either to move or breathe.

If those clever young medical men whose physique is so fine, and whose minds are so expansive and free, could but be put down in tens of thousands all over India, China, and Africa, what splendid centres of civilisation they would make. They are just the sort of men to carry Western life blood through the arteries of the anæmic and languid East. It is a marvel that they do not push their way into those great open markets for cultured work. It is specially surprising that young medical men do not seek the medical work that is so abundant in uncivilised and half-civilised countries. The young doctor is generally bursting with eagerness to try his newly-acquired knowledge and skill on actual and serious cases. He cannot get such cases in this country: they are already in the hands of practitioners older and more competent than himself. But in the countries outside civilisation clinical material of every kind is overwhelming in its abundance. A competent medical authority thus summarises the condition of the sick in most of the countries which are outside the influence of Western civilisation: "Think," he says, "of the sad condition of the sick in uncivilised lands. Denied proper food, often literally starved to death, bled to excess, tortured with needle and cautery, left neglected, and often naked on the bare ground. Think of the neglected eye diseases, ending in blindness; of the neglected bone disease, enfeebling and crippling; of the malignant tumours, disfiguring, torturing, and killing. Your skill, if consecrated to their service, would give sight to many who will otherwise remain blind, would restore strength and usefulness to diseased limbs, and would give back bread-winners to their families." We might have expanded this statement a hundredfold without exhausting the bare facts of the case.

Is it not something of a miracle that the great missionary societies have made so little use of the beneficent and civilising skill of trained and qualified medical men? One would have thought that a young and enthusiastic doctor would be the very man to put down in a large and populous district in China or Africa. True, he might not teach dogma very well, but he would heal the sick; and he would teach his patients to be honest and truthful, and to try to love one another. Moreover, he would always be among them as a living example of Western manliness, Western virtues, and Western trained and practised skill. If the missionary societies were half as wise as they should be, they would offer, not only working careers, but adequate pay and pensions, on the lines of the public services, to the highest class of men which are to be found at our medical schools. And if those young men were altogether as enterprising and as generous as they should be, they would seek to devote their lives to the great and noble work which is waiting to be done in uncivilised and half-civilised countries, even though they had to submit to the somewhat cramping conditions imposed by the missionary societies. But in truth, all classes of us who feel that in England the workers are more than the work, whilst in our large dependencies the work is more than the workers, must give earnest consideration to the subject in order that where the work is greatest and most urgent, there the workers may be sent in ever-increasing numbers.

